# P-1014. Blastemia is a Poor Prognostic Factor in Leukemia Patients with Culture-documented Invasive Pulmonary Aspergillosis

**DOI:** 10.1093/ofid/ofae631.1204

**Published:** 2025-01-29

**Authors:** Sung-Yeon Cho, Sebastian Wurster, Ying Jiang, Russell E Lewis, Takahiro Matsuo, Nathaniel D Albert, Guillermo Garcia-Manero, Dimitrios P Kontoyiannis

**Affiliations:** Division of Infectious Diseases, Department of Internal Medicine, College of Medicine, The Catholic University of Korea, Seoul, Korea, Seoul, Seoul-t'ukpyolsi, Republic of Korea; The University of Texas MD Anderson Cancer Center, Houston, Texas; The University of Texas MD Anderson Cancer Center, Houston, Texas; University of Bologna, Bologna, Emilia-Romagna, Italy; The University of Texas MD Anderson Cancer Center, Houston, Texas; The University of Texas MD Anderson Cancer Center, Houston, Texas; MD Anderson Cancer Center, Houston, Texas; The University of Texas MD Anderson Cancer Center, Houston, Texas

## Abstract

**Background:**

Severe and prolonged neutropenia is a well-known risk factor for invasive pulmonary aspergillosis (IPA) and is also associated with poor outcomes. Given the increasing frequency of IPA patients (pts) with relapsed/refractory leukemia, we hypothesized that high peripheral blood (PB) blast burden (blastemia) might be an additional cause of antifungal immune failure, even in the absence of neutropenia. Therefore, we evaluated the prognostic significance of blastemia in leukemia pts with IPA.

Definitions of D- and B-indices

**Figure:**
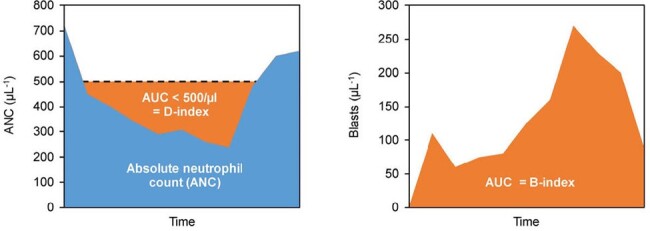

**Methods:**

We retrospectively reviewed the medical records of adult pts with acute leukemia or myelodysplastic syndrome diagnosed with culture-positive proven/probable IPA at MD Anderson Cancer Center in 2011–2022. Blast-index (B-index) and neutropenia index (D-index) were defined as the area-under-the-curve of PB blast counts and absolute neutrophil counts < 500/µL, respectively, from admission to IPA diagnosis (Fig 1). Predictors of 42-day (42d) survival after IPA diagnosis were determined by univariate statistics and a logistic regression model.

Four-group survival curve analysis according to blastemia and neutropenia status at IPA diagnosis (Mantel-Cox log-rank test)

**Figure:**
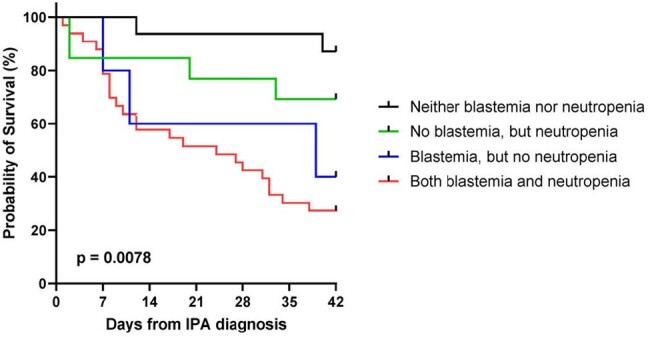

**Results:**

Among the 74 included pts, 50 (68%) had neutropenia and 42 (57%) had blastemia at IPA diagnosis. Age ≥65 years (54% *vs*. 29%, *p*=0.035), active leukemia (95% *vs*. 74%, *p*=0.011), neutropenia (82% *vs*. 53%, *p*=0.008), and blastemia before IPA diagnosis (74% *vs*. 38%, *p*=0.002) were more common in 42d non-survivors than in 42d survivors. This observation was corroborated by four-group survival analysis according to blastemia and neutropenia status (*p*=0.008, **Fig 2**). Logistic regression analysis identified previous antifungal exposure (aOR 22.30, *p*=0.012), B-index before IPA diagnosis ≥90 (aOR 5.65, *p*=0.010), and D-index ≥650 (aOR 4.33, *p*=0.025) as significant independent predictors of 42d mortality after IPA diagnosis. B-index ≥90 was also a significant predictor of antifungal treatment failure (aOR 7.37, *p*=0.004), which was in turn associated with 42d mortality (*p*=0.002).

**Conclusion:**

Blastemia is common in contemporary leukemia pts with IPA and is a strong and significant independent risk factor for poor IPA outcome, regardless of neutropenia status. This observation has implications for stratification of leukemia pts in future mycology trials and underscores the need to incorporate active leukemia and blastemia in preclinical IPA models.

**Disclosures:**

**Sebastian Wurster, MD, MSc**, Astellas Pharma: Grant/Research Support|Gilead Sciences: Grant/Research Support **Dimitrios P. Kontoyiannis, MD**, AbbVie: Advisor/Consultant|Astellas Pharma: Advisor/Consultant|Astellas Pharma: Grant/Research Support|Astellas Pharma: Honoraria|Cidara Therapeutics: Advisor/Consultant|Gilead Sciences: Advisor/Consultant|Gilead Sciences: Grant/Research Support|Gilead Sciences: Honoraria|Knight: Advisor/Consultant|Merck: Advisor/Consultant|Scynexis: Advisor/Consultant

